# Personal Exposures to Traffic-Related Air Pollution and Acute Respiratory Health among Bronx Schoolchildren with Asthma

**DOI:** 10.1289/ehp.1002653

**Published:** 2011-01-07

**Authors:** Ariel Spira-Cohen, Lung Chi Chen, Michaela Kendall, Ramona Lall, George D. Thurston

**Affiliations:** 1 Nelson Institute of Environmental Medicine, New York University School of Medicine, Tuxedo, New York, USA; 2 European Centre for Environment and Human Health, Peninsula College of Medicine and Dentistry, University of Exeter, The Knowledge Spa, Truro, Cornwall, United Kingdom

**Keywords:** air pollution, asthma, children’s health, diesel, elemental carbon, personal monitoring traffic, PM_2.5_

## Abstract

**Background:**

Previous studies have reported relationships between adverse respiratory health outcomes and residential proximity to traffic pollution, but have not shown this at a personal exposure level.

**Objective:**

We compared, among inner-city children with asthma, the associations of adverse asthma outcome incidences with increased personal exposure to particulate matter mass ≤ 2.5 μm in aerodynamic diameter (PM_2.5_) air pollution versus the diesel-related carbonaceous fraction of PM_2.5_.

**Methods:**

Daily 24-hr personal samples of PM_2.5_, including the elemental carbon (EC) fraction, were collected for 40 fifth-grade children with asthma at four South Bronx schools (10 children per school) during approximately 1 month each. Spirometry and symptom scores were recorded several times daily during weekdays.

**Results:**

We found elevated same-day relative risks of wheeze [1.45; 95% confidence interval (CI), 1.03–2.04)], shortness of breath (1.41; 95% CI, 1.01–1.99), and total symptoms (1.30; 95% CI, 1.04–1.62) with an increase in personal EC, but not with personal PM_2.5_ mass. We found increased risk of cough, wheeze, and total symptoms with increased 1-day lag and 2-day average personal and school-site EC. We found no significant associations with school-site PM_2.5_ mass or sulfur. The EC effect estimate was robust to addition of gaseous pollutants.

**Conclusion:**

Adverse health associations were strongest with personal measures of EC exposure, suggesting that the diesel “soot” fraction of PM_2.5_ is most responsible for pollution-related asthma exacerbations among children living near roadways. Studies that rely on exposure to PM mass may underestimate PM health impacts.

Adverse respiratory health outcomes in published traffic proximity–exposure studies include increased prevalence of asthma (McConnell et al. 2006; Nicolai et al. 2003), wheezing (Gauderman et al. 2005; Nordling et al. 2008), recurrent respiratory illnesses (Kim et al. 2004), decreased lung function (Balmes et al. 2009; Brunekreef et al. 1997; Kan et al. 2007), increased lung inflammation markers (Dales et al. 2008; Holguin et al. 2007), and increased medical visits and hospital admissions for asthma (Chang et al. 2008; English et al. 1999). Increased immunoglobulin E (IgE) production from controlled exposure to diesel exhaust particles (DEP) (Diaz-Sanchez et al. 1997) provides mechanistic support for the role of DEP in the associations of adverse respiratory health with traffic indicators. The presence of elemental carbon (EC) in the lung has also been directly associated with adverse effects (Kulkarni et al. 2006), and increased EC concentrations have been associated with increased exhaled nitric oxide—a marker of airway inflammation (Delfino et al. 2006)—supporting the hypothesis that it is exposure to DEP that causes the associations of traffic with asthma exacerbations. Personal measurements of vehicle-related particle exposures and acute respiratory health outcomes are currently lacking in most studies of this issue.

Bronx County has among the highest incidences of asthma emergency department (ED) visits and hospital discharges both in New York City (NYC) and throughout New York State (New York State Department of Health 2009). The South Bronx area of Bronx County is particularly affected, with children’s asthma hospitalization rates several times higher in the South Bronx than in other NYC areas (Claudio et al. 2006). The South Bronx provides an ideal context in which to investigate the effects of traffic-related air pollution on children with asthma because large numbers of children with asthma live and attend schools in close proximity to highways and businesses that rely on high volumes of truck traffic. Large volumes of heavy truck traffic pass through the South Bronx along several major highways, as well as to and from a quarter of NYC’s waste transfer facilities (Naphtali et al. 2007), the Bronx Terminal, and Hunts Point wholesale fish, flower, and produce markets.

Although EC is not a unique marker for DEP exposure in all cases, our previous exposure assessment work in the study area has shown that EC concentrations are a reliable indicator of truck traffic–generated pollution in this inner-city locale, for which we found evidence that > 90% of the Bronx EC can come from diesel exhaust (Spira-Cohen et al. 2010). New York University’s (NYU) prior exposure assessment work in the study area (Maciejczyk et al. 2004) also showed that variations in ground-level BC concentrations in the South Bronx were related to local truck traffic density.

Although levels of other pollutants have shown a significant decline in NYC in the last decade, EC levels have not shown the same decline (Narvaez et al. 2008). In the previously published exposure assessment findings of this study, we found that children living closer to a highway had progressively higher mean personal EC exposures and that concentrations of EC varied with hourly truck traffic measurements near the most heavily trafficked school sampling site (Spira-Cohen et al. 2010). By collecting personal monitoring data from a group of children with asthma in the South Bronx, in this analysis we are able to directly compare the health effects from exposure to particulate matter ≤ 2.5 μm in aerodynamic diameter (PM_2.5_) versus the diesel “soot” EC-related fraction of PM_2.5_. We therefore hypothesized that associations of adverse respiratory health effects with the diesel fraction (as indicated by a metric of EC) will be stronger and more consistent than with PM_2.5_ total mass. We tested this hypothesis more definitively than past studies have done by using daily personal PM_2.5_ measurements collected in a group of inner-city children with asthma.

The panel study design we employed in this research allows for each subject to act as his or her own control, whereby low personal exposure days are compared with high-pollution days in the same subject. Therefore, factors that vary among individuals remain consistent over time, and the health effects of daily pollutant levels can be quantified and compared.

## Materials and Methods

Our panel of study subjects included 40 fifth-grade elementary school children (10–12 years of age) who were either doctor-diagnosed with asthma or had experienced asthma attacks while at school for which the child visited the school’s nurse. Subjects were referred to the study by the school’s nurse as suffering from asthma. On enrollment, baseline asthma questionnaires adapted from the ISAAC (International Study of Asthma and Allergies in Childhood) (Asher et al. 1995) were completed by the child and his or her parent or guardian to determine the asthma history of each subject and to confirm the child’s asthma status. All questionnaires were available in Spanish, so all Hispanic parents who were not comfortable in English were given a Spanish version. These questionnaires were administered by a bilingual staff person from NYU. No participants came from homes where parents were not proficient in either English or Spanish. Participants were volunteers, and parents/guardians gave written informed consent for their child. The study protocol was approved by the NYU Institutional Review Board and meets all U.S. requirements for human subjects research.

During approximately a 1-month sampling period at each school, personal and outdoor school-site monitoring data were collected at four elementary schools: two schools adjacent to and two schools farther away from a highway. The school with the highest traffic impact was located 173 feet from the Major Deegan Expressway [annual average daily traffic (AADT), 112,051 vehicles/day], and the school with the lowest traffic impact was located 2,419 feet from the Bruckner Expressway (AADT, 100,230 vehicles/day) (New York State Department of Transportation 2010). Local street traffic was minimal at the two schools farther away from highways; however, all schools sampled experienced some amount of local traffic from the various truck routes in the area (e.g., leading to and from the Hunts Point produce markets).

Daily health outcomes and personal PM_2.5_ exposures were collected simultaneously for 10 subjects from each of the four South Bronx schools. One school was sampled during each sampling session: spring 2002, spring 2004, fall 2004, and spring 2005, for a total of 4 schools sampled. Sampling took place during the spring or fall seasons to minimize effects of summer haze air pollutants such as ozone (O_3_) and acidic aerosols, and because the schools were in session at those times. Personal exposures were collected using a rolling backpack with air pollution monitoring equipment attached to the upper handle as close as possible to the breathing zone. The subjects took the backpack along with them 24 hr/day during the sampling period. Daily time–activity diary data were recorded every 15 min, including nearby cooking and smoking. Any missing diary data from the previous day were filled in retrospectively with NYU staff at the school. In the case of school absences, NYU staff visited children in their homes to change filters and check diary entries whenever possible. The diligent follow-up by NYU staff led to a data collection rate of close to 100% for weekday diary data. Data from motion sensors and the activity diaries were reviewed daily to confirm that the child took the backpack along during all recorded activities.

The subjects’ backpack samplers collected 24-hr integrated weekday samples of PM_2.5_ and the EC portion. Simultaneously collected school-site continuous PM_2.5_ (TEOM 1400a; Thermo Electron Corp., East Greenbush, NY, USA) and BC (aethalometer model AE-21; Magee Scientific, Berkeley, CA, USA) were averaged for 0900 hours to 0900 hours weekdays to match the personal measurements. Twice daily (0700 hours to 1400 hours, 1400 hours to 0700 hours) weekday PM_2.5_ filter samples (ACCU sampler; R&P, Thermo Electron Corp.) were collected and subjected to X-ray fluorescence analysis for sulfur determination. Details on sampling instruments and methods have been published previously (see Spira-Cohen et al. 2010, online supplement). Hourly concentrations of nitrogen oxides (Columbia Scientific, Austin, TX, USA), O_3_ (Thermoenviron, Hopkinton, MA, USA), and sulfur dioxide (SO_2_; Monitor Labs, Englewood, CO, USA) were also monitored at the schools. Background measurements of PM_2.5_ from a New York State Department of Environmental Conservation rooftop monitor on one of the four schools (PS154) were also available for comparison.

Subjects recorded respiratory symptoms three times daily by rating their symptoms on a scale of 0 to 5, with 0 indicating no symptoms present and 5 indicating a hospital or asthma clinic visit, following the Inner City Asthma Study (ICAS) protocol (Mortimer et al. 2002). Daily symptom severity indices were created for wheeze, cough, and shortness of breath by adding morning and afternoon daily symptom scores, and a composite of symptom severity score (wheeze + cough + shortness of breath) was also computed to account for the fact that types of symptoms may differ for different children. Weekday morning and afternoon lung function measurements were administered under the supervision of the NYU researchers at the school following American Thoracic Society guidelines for spirometry (Miller et al. 2005) using portable Asthma Monitor 1 spirometers (Jaeger Instruments, Hoechberg, Germany); thus, only acceptable spirometry maneuvers were included in the data set. Measurements were downloaded daily, and anomalous data points from unsupervised tests were flagged. Afternoon (1500 hours) peak expiratory flow (PEF) and forced expiratory volume in 1 sec (FEV_1_) were investigated in this analysis because NYU researchers were present during these maneuvers, and because these measures of airway obstruction are traditionally used for diagnosis of asthma severity (Sawyer et al. 1998).

Mixed-effects models have been used successfully for longitudinal studies of exposure–health effects (Buckeridge et al. 2002; Delfino et al. 2004, 2006; Trenga et al. 2006) and were applied here using an *a priori* subject-specific random intercept term, as used in similar panel studies, which effectively controls for the nonindependent nature of observations within the same subject. A generalized form of the mixed model (see Bailey and Alimadhi 2007) was applied to the symptom severity indices, which followed a Poisson distribution. A standard linear mixed model (Pinhiero and Bates 2000) was applied to the lung function metrics. The models were run in S-Plus (version 6.1; Insightful Corporation, Seattle, WA, USA), using the “MASS” and “nlme” modeling libraries for symptom and lung function models, respectively. School was included as a covariate in final symptom models. Height, sex, and temperature were included in final lung function models. The addition of school as a covariate also accounted for any potential seasonally related confounding. Because school was not a significant covariate in the lung function models, this term was not included in the final regression models. All models included an AR-1 autocorrelation term to address the time-series nature of the data. Health associations with personal and school-site measurements were evaluated with same-day concentrations, 1-day lag concentrations, and an average of the same day and previous day’s pollution concentrations (2-day average). Longer lags were not investigated because of the availability of weekday personal data only, because it was not feasible to follow up with all children in their homes on weekends. All results are reported using the 5th to 95th percentile pollutant concentrations as an index of effect size on a clean day versus a polluted day.

## Results

Table 1 lists descriptive statistics of the study subjects [for school-specific descriptive statistics, see Supplemental Material, Table 1 (doi:10.1289/ehp.1002653)]. Of the 40 children enrolled in this panel, 22 were male and 18 were female. The overall mean age was 11 years, and all attended the fifth grade. All children were African American or Hispanic of Caribbean descent. Most children spent most of their time indoors (mean, 89%), came from nonsmoking households, and lived near their school, arriving at school on foot. Thirty-five of the 40 children had used medication sometime within the preceding 12 months, and 16 (of 36 reporting) had visited an ED or were hospitalized for asthma symptoms within the previous year. Thirty-one subjects (of 36) reported using a “rescue” inhaler or nebulizer. Nine subjects reported using some form of controller medication.

### Symptom–PM analysis

**Figure 1:**
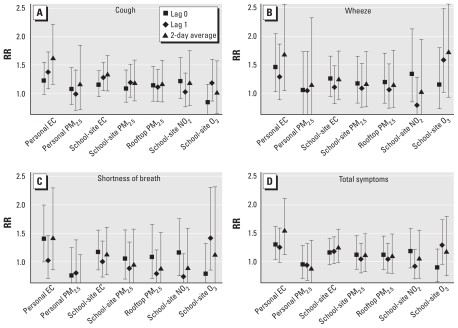
Relative risks (RR) (with 95% CIs) of cough (*A*), wheeze (*B*), shortness of breath (*C*), and total symptom (*D*) severity scores associated with the various personal and outdoor school-site particle and gas exposure measurements. Lag EC models included predicted Sunday values from subject-specific personal-school site EC regression coefficients [except for two subjects with outlying relationships with the school-site monitor (*r* < 0.1)]. Because of poor correlations of personal with school-site PM_2.5_, this was not feasible for PM_2.5_ models. Personal EC: *n* = 563 for the same day; *n* = 571 for lag 1 day; *n* = 516 for 2-day average. Personal PM_2.5_: *n* = 556 for the same day; *n* = 465 for lag 1-day; *n* = 419 for 2-day average. All school-site models: *n* = 625 for the same day; *n* = 617 for lag 1 day; *n* = 607 for 2-day average.

Descriptive statistics of personal exposure measurements are reported in the Supplemental Material (doi:10.1289/ehp.1002653). Relative risks (RRs) for symptom severity indices were at or close to statistical significance for same-day personal EC exposure concentrations. We found an increased risk of cough [RR = 1.23; 95% confidence interval (CI), 0.99–1.54], wheeze (RR = 1.45; 95% CI, 1.03–2.04), shortness of breath (RR = 1.41; 95% CI, 1.01–1.99) and total symptom (RR = 1.30; 95% CI, 1.04–1.61) severity per 3.0 μg/m^3^ EC (5th–95th percentile) concentration. We also found an increased risk for cough severity (RR = 1.37; 95% CI, 1.09–1.72) with 1-day lag personal EC concentrations. Elevated risk of cough, wheeze, and total symptom severity was significant with 2-day average concentrations (cough: RR = 1.61; 95% CI, 1.17–2.21; wheeze: RR = 1.67; 95% CI, 1.05–2.66; total symptoms: RR = 1.54; 95% CI, 1.13–2.10) (Figure 1). We did not find an increased risk of any of the symptom outcomes from increased exposure to personal PM_2.5_ with same-day, 1-day lag, or 2-day average concentrations.

An increased risk of cough severity (RR = 1.28; 95% CI, 1.06–1.54) was significantly associated with 1-day lag school-site EC. With a 2-day average concentration, school-site EC was also associated with an elevated risk of cough severity (RR = 1.32; 95% CI, 1.05–1.66) and total symptom severity (RR = 1.24; 95% CI, .99–1.56) (Figure 1). We found no statistically significant relationships of increased risk of symptoms with any of the other school-site PM measurements, although RR effect estimates were all > 1.0. Except for shortness of breath, RR estimates were larger with a 2-day average personal EC concentration than with either 1-day lag or same-day exposure concentrations, although CIs are also somewhat larger. Correlations of same-day personal concentrations with 1-day lag school-site concentration were relatively low for both EC (*r* = 0.27) and PM_2.5_ (*r* = 0.17).

### Lung function–PM analysis

Although none of the lung function metrics showed associations below the *p* < 0.05 significance threshold with any of the exposure variables evaluated in the mixed model, several relationships were nearly significant (*p* < 0.10) for decreased lung function versus increased personal pollution exposure (Table 2). We found a decrease of 9.13 L/min (95% CI, −19.02 to 0.86 L/min; *p* = 0.07) with increased personal EC, representing an average decline in PEF of approximately 3.5%. Although we also found an association with FEV_1_ decline with increased personal EC exposure, this association was not close to statistical significance.

Decrements in both PEF and FEV_1_ were associated with increased personal PM_2.5_ exposure, with a decrease in PEF of 9.40 L/min (95% CI, −20.43 to 2.08 L/min) and in FEV_1_ of 0.06 L (95% CI, −0.14 to 0.01 L), both representing an average decline of approximately 3.4%, with the association with FEV_1_ close to statistical significance.

We found no significant relationships between lung function decline and any of the school-site PM or gaseous measurements (Table 2).

### Sensitivity analyses

To test whether overall results were driven by individual subjects with unusual characteristics, we excluded potentially outlying subjects and reevaluated the mixed model results. The PEF decrement effect estimate increased slightly from −9.1 L/min to −11.7 L/min, and the marginal significance of the pollution reached the *p* < 0.05 significance level, when we excluded one outlier subject with extreme personal exposures. The FEV_1_ decline associated with personal PM_2.5_ decreased from −0.06 to −0.05, and the confidence band widened, when this subject was excluded. Overall, models were robust to exclusion of other potentially outlying subjects, including asymptomatic subjects, subjects with unusually low lung function measurements, and subjects with higher than average personal exposures.

We also tested whether results may have been driven by individual schools by excluding each school from the overall model. When we excluded School MS302, a school farther from a highway (1,216 feet), from the PEF model with personal EC, the marginal significance of the EC pollution increased to *p* = 0.43, although the effect estimate remained consistently negative, diminishing by one-third. We found a maximum change in coefficient size of less than one-third when we excluded any of the other schools from the PEF model, and *p*-values remained < 0.10. *p*-Values also increased when schools were excluded from FEV_1_ models with personal PM_2.5_, but the magnitude of the effect estimate was robust to exclusion of schools.

Using percent predicted standardized lung function metrics also produced similar effect estimates. Models of a transformed lung function outcome, computed by taking the difference from each child’s mean value, showed stronger associations for personal EC with PEF (*p* < 0.02) but weaker, less significant associations for personal PM_2.5_ with both FEV_1_ and PEF.

We included school as a covariate in final symptom models because it was significant in all symptom models except wheeze. It was not a significant covariate in the lung function models and did not change the lung function–pollutant effect estimates, and was therefore not included in those final models. An indicator variable for schools closer to a highway was significant only in models of shortness of breath (*p* < 0.05) and marginally significant in models of total symptoms (*p* = 0.09), with higher symptoms at schools closer to the highways.

Symptom results remained robust even when subjects and schools were excluded from the models, although models of shortness of breath were least robust. Results were robust to exclusion of the school sampled in the fall season. The addition of indicator variables representing day of week, respiratory infections (evening presence/absence), and school absences did not confound any of the significant associations.

To determine whether model results were driven by subjects with more severe asthma, as indicated in our data by reporting an ED visit within the preceding 12 months, we investigated this group separately from the group reporting no ED visit. We found no significant difference in risk between the two subgroups, although relative risks were slightly higher in the group having visited an ED. For lung function, the EC–PEF association was larger in the subjects who had not visited an ED; however, the two groups’ estimates were also not statistically different from each other.

### School-site gaseous pollutant analysis

We also investigated the roles of O_3_ [a secondary gaseous pollutant known to acutely affect asthma (Gent et al. 2003; Thurston and Bates 2003; Thurston et al. 1997; Trasande and Thurston 2005)], nitrogen dioxide [NO_2_; a general indicator of fresh traffic exhaust also previously associated with asthma exacerbations (Delfino et al. 2006)], and SO_2_ (a known acute bronchoconstrictor [see U.S. Environmental Protection Agency (EPA) 2008]) to consider potential confounding of the PM–health associations by gaseous copollutants.

The maximum outdoor daytime (6-hr average) O_3_ concentration during school hours (0900 hours to 1500 hours) was 73 ppb during the study (mean, 23 ppb). We found a significant association of increased 6-hr average O_3_ with increased risk of wheeze severity with a 1-day lag. This association with 1-day lag O_3_ was robust to exclusion of the highest O_3_ day (O_3_ maximum decreased to 50 ppb after exclusion), as well as exclusion of the school experiencing days with the highest O_3_ levels (O_3_ maximum decreased to 40 ppb after exclusion). However, in a copollutant model of same-day EC and 1-day lag O_3_ with wheeze, EC remained a significant exposure factor.

Six-hour average NO_2_ (0900 hours to 1500 hours) was not significantly associated with any of the health outcomes investigated, although risk of symptoms was greater than 1.0 with same-day concentrations. In a copollutant model of NO_2_ and EC, EC remained the significant exposure factor. Peak morning SO_2_ concentrations were also significantly associated with symptom outcomes. Peak morning SO_2_ was associated with increased risk of both cough (RR per 30 ppb SO_2_ = 1.42; 95% CI, 1.15–1.76) and wheeze (RR = 1.56; 95% CI, 1.11–2.19). SO_2_ and EC contributed equally in a copollutant model.

School-site EC was not highly correlated with NO_2_ (*r* = 0.36) or O_3_ (*r* = 0.25) at the time intervals investigated in the health effects analysis. On an hourly basis, NO_2_ showed much higher correlations with school-site EC (*r* = 0.60), and SO_2_ was also moderately correlated (*r* = 0.45) during these study sampling periods (i.e., during spring and fall). Thus, although O_3_ and SO_2_ were also associated with some asthma morbidity metrics, they did not displace EC in effects analyses.

### School-site sulfur analysis

**Figure 2:**
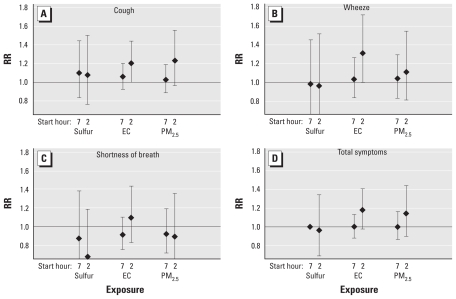
Relative risks (RR) (with 95% CIs) of cough (*A*), wheeze (*B*), shortness of breath (*C*), and total symptom (*D*) severity scores associated with the school-site integrated measurements of sulfur, EC, and PM_2.5._ Measurements were taken from 0700 hr to 1400 hr (start hour = 7) and from 1400 hours to 0700 hours (start hour = 2). Sulfur and PM_2.5_: *n* = 615 for 0700 hours to 1400 hours; *n* = 605 for 1400 hours to 0700 hours. EC: *n* = 635 for 0700 hours to 1400 hours; *n* = 625 for 1400 hours to 0700 hours. RR of total symptoms for 0700 hours to 1400 hours sulfur = 1.0 (95% CI, 0.99–1.00).

We found significant elevated risks of symptoms with EC concentrations but not with total PM_2.5_ or the sulfur fraction in models separately evaluating the school-site measurements of PM_2.5_, EC, and sulfur from the same filter. We found increased risk of cough and wheeze severity with school-site filter EC concentrations measured from 1400 hours the previous day to 0700 hours the next morning (weekdays), but not with same-day school-hour filter EC (0700 hours to 1400 hours weekdays). We found nearly significant positive associations for increased risk of cough severity with school-site filter PM_2.5_ (1400 hours to 0700 hours). The sulfur fraction of the PM on the school-site filters was not associated with an increase in any of the symptom outcomes for either of the integrated time periods (Figure 2), and school-hour weekday sulfur concentrations were not associated with either of the afternoon lung function metrics. Thus, school-site sulfur concentrations were not confounders of the EC–asthma symptom associations.

## Discussion

The present analyses indicate that personal exposure to the EC portion of PM_2.5_, which in our exposure assessment analysis was strongly affected by diesel exhaust emissions in this locale (Spira-Cohen et al. 2010), is most associated with several adverse respiratory outcomes in a group of inner-city elementary school children with asthma. Symptom severity was the most complete health record and yielded the most consistent results, showing a significant increase in risk with increasing exposure to EC concentrations. Associations between adverse symptoms and PM_2.5_ mass, measured both via the personal backpacks and at the school site, were, in general, in the same direction as for EC but had larger *p*-values.

Measures of BC soot have previously been linked specifically with decreased lung function (Suglia et al. 2008) and with markers of lung inflammation, such as exhaled nitric oxide (Delfino et al. 2006; Kulkarni et al. 2006). Among healthy children, reduced lung function has been associated with carbon content in the lungs (Kulkarni et al. 2006), and EC concentrations with deficits in lung growth in children 10–18 years of age (Gauderman et al. 2004). A study of very young children in NYC found associations of cough during the cold/flu season with central-site EC concentrations, but not with PM_2.5_ (Patel et al. 2009). Short-term exposures to DEP also led to the enhancement of allergic inflammation (Diaz-Sanchez et al. 1997; Pourazar et al. 2005). The findings of the present study provide further evidence to support the hypothesis that previous associations of asthma exacerbations with traffic are due to exposure to the DEP fraction of PM. EC is also associated with very small PM (< 1 μm) with larger surface area (U.S. EPA 2004).

The strongest and most consistent associations we found were for personal EC exposures with increased risk of asthma-related symptoms, which were statistically significantly associated with increased same-day personal EC concentrations. We found not only strong associations with same-day concentrations, but also a significant increase in risk of cough with a 1-day lag and the largest relative risks for wheeze and cough with a 2-day average EC concentration. These associations suggest a possible delay in the onset of symptoms and/or possible cumulative effects because triggers for asthma reactions can contribute to both immediate and late-phase responses. Our finding of significant lag effects with stationary-site monitors is consistent with a similar study showing significant effects for exposure to EC with exhaled nitric oxide in children with asthma: Delfino et al. (2006) also found significant associations with same-day and 2-day moving average concentrations of personal EC, but only with 1-day lag and 2-day moving average concentrations for central-site EC measurements.

Although previous studies have investigated effects on PEF because of its ease in collection and its correlation with FEV_1_, our study had the advantage of investigating both of these lung function metrics directly measured simultaneously. Our results support those of other studies finding associations of lung function change with exposure to PM pollution: both PM_2.5_ mass (Delfino et al. 2004, 2008; Suglia et al. 2008) and the EC fraction (McCreanor et al. 2007; Suglia et al. 2008). Reductions in lung function found in this work may underestimate the full effects because during the times children experienced the most severe symptoms, they were unable to blow into the spirometer. For instance, on five afternoon occasions, lung function measurements were missing when the same child recorded substantial symptoms. Furthermore, we did not obtain sufficient data on rescue medication, which may also have masked lung function effects. Increased frequency of as-needed medication use has itself been associated with exposure to air pollution (Gent et al. 2003; Rabinovitch et al. 2006; Thurston et al. 1997; Trasande and Thurston 2005; Von Klot et al. 2002).

We also investigated the effects of copollutants. We found a significant association of increased risk of wheeze with O_3_ concentration at 1-day lag, even when the highest O_3_ concentrations were excluded. Risks of other symptoms were also elevated, but were not statistically significant. Associations of both same-day and lag effects of O_3_ concentrations with adverse respiratory health are widely documented in the literature (Thurston and Bates 2003; U.S. EPA 2008). SO_2_ gas, a marker of fresh diesel exhaust in the South Bronx (sampling took place in warm, nonheating seasons and before regulations mandating low-sulfur diesel), was also significantly associated with increased risk of symptoms. Although the associations we found with O_3_ and SO_2_ may have their own implications for respiratory health, they did not confound the significant symptom associations found with EC in copollutant models. In addition, there is recent evidence of a biological interaction of exposure to DEP and O_3_ in the lung (Bosson et al. 2008). Furthermore, the association we found with SO_2_ is consistent with a role for diesel in our findings with EC, because the copresence of diesel SO_2_ with DEP may potentiate the respiratory effects of the diesel aerosols.

### Study limitations

A significant limitation of this study is the relatively small number of subjects studied because feasibility issues allowed us to enroll only 40 study participants. Future studies enrolling more subjects may help narrow the uncertainty around the effect estimates we found in the present study, as well as provide the increased power necessary to investigate differences in potential effect modifiers, such as asthma severity or school distance from a highway. Although our original intent in the study was to investigate differences between schools, this was not possible because of the lack of simultaneous school-site measurements and observed similarities in exposure levels among all schools. Because all schools were located in a dense urban locale, schools located farther away from highways were still affected by local traffic (see Spira-Cohen et al. 2010).

An additional limitation of this study was the lack of adequate data on daily medication use. Many of the children who reported some use of controller medications took them as needed, rather than on a regular basis, which we suspect may reflect findings of underuse of controller medications in populations similar to that of our study (i.e., blacks and Hispanics of lower socioeconomic status) (e.g., Finkelstein et al. 2002). Because the daily medication habits of the participants did not change before or during the study, the observed associations are not likely to be attributable to confounding by medication use.

Although our symptom score collection methodology may have the potential for reporting bias, we do not expect this bias to confound our results because the subjects were unaware of their personal pollution measurements. Similarly, all subjects had the same potential for measurement error from the backpack samplers, which were attached to the backpack as close as possible to the breathing zone (waist height or above).

A further limitation of this study is that our EC measurements could indicate other properties of traffic-related air pollution that are correlated with EC. We acknowledge that other pollutants that we did not measure in this study, such as ultrafine particle concentrations, kicked-up road dust, and/or particles from tire wear, cannot be ruled out as potential confounders of exposure.

## Conclusion

A major strength of this study was the ability to obtain daily measures of personal exposure to EC (rather than using PM_2.5_ mass, distance from roadway indices, or central-site data). Past traffic-related air pollution studies investigating health effects have relied mainly on central-site monitoring data, modeled exposure variables, or employed proximity to roadway as an exposure metric. In addition, this study found similar, albeit weaker, associations using school-site monitoring for EC, suggesting that school-site stationary measurements of EC may be representative of average daily personal exposures across the study participants in this dense urban setting. However, we found the strongest health–EC associations with the more accurate personal measure of “actual” exposure.

Using personal measurements, our findings more definitively confirm those of other recent urban exposure–asthma studies that have also pointed to the carbonaceous fraction of the PM, rather than total PM_2.5_ mass, as showing stronger associations with adverse respiratory health. Therefore, exposure–health effects studies that rely on exposure measures of PM mass from central-site monitors may be underestimating health relationships with individual components of the PM.

## Correction

In Results (“Symptom–PM analysis”), risks and 95% CIs were presented as percentages in the original text published online. They have been converted to RRs here.

## Figures and Tables

**Table 1:** Subject characteristics and descriptive statistics.

Characteristic	All schools
Age [years (mean)]	11
Sex (*n*)	
Male	22
Female	18
Subjects owning cat/dog (*n*)	8
Hospitalized or visited ED for asthma in previous 12 months (*n*/total)^a^	16/35
Rescue inhaler or nebulizer (*n*/total)	31/36
Mean afternoon PEF < 80% predicted (*n*/total)	5/40
Mean afternoon FEV_1_ < 80% predicted (*n*/total)	14/40
PEF, morning [L/min (mean ± SD)]^b^	259 ± 54
PEF, afternoon [L/min (mean ± SD)]^b^	271 ± 49
FEV_1_, morning [L (mean ± SD)]^b^	1.73 ± 0.37
FEV_1_, afternoon [L (mean ± SD)]^b^	1.78 ± 0.33
Daily symptom score [median (minimum–maximum) *n* subjects with positive symptom score per day]^c^	
Cough	6 (0–10)
Wheeze	3 (0–6)
Shortness of breath	3 (0–8)
Total symptoms	7 (0–10)^c^

a Data missing for five subjects.

b The afternoon maneuver closest to 1500 hours (of afternoon measurements from 1200 hours to 1800 hours) was used for the analysis. If more than one measurement was taken within an hour of 1500 hours (from 1400 hr to 1600 hours) an average of the values was computed.

c For example, a given day during the study had a median of 7 of the 10 subjects having a positive symptom score, with a minimum of 0 subjects and a maximum of 10 subjects exhibiting symptoms.

**Table 2:** Mixed-model estimates of lung function decrements associated with personal and school-site pollutants.

Exposure (pollution increment)^a^	Health outcome^b^	Effect (95% CI)
Personal EC (3.0 μg/m^3^), *n* = 434	PEF^c^	−9.13 (−19.13 to 0.86)*
	FEV_1_^d^	−0.02 (−0.09 to 0.04)

Personal PM_2.5_ (55 μg/m^3^), *n* = 431	PEF	−9.40 (−20.43 to 2.08)
	FEV_1_	−0.06 (−0.14 to 0.01)*

School-site EC (3.0 μg/m^3^), *n* = 454	PEF	−4.58 (−14.01 to 4.85)
	FEV_1_	0.01 (−0.04 to 0.07)

School-site PM_2.5_ (22 μg/m^3^), *n* = 454	PEF	−3.06 (−15.11 to 8.98)
	FEV_1_	−0.02 (−0.09 to 0.06)

Rooftop PS154 PM_2.5_ (24 μg/m^3^), *n* = 454	PEF	−0.11 (−12.48 to 12.32)
	FEV_1_	−0.02 (−0.10 to 0.06)

NO_2_ (60 ppb), *n* = 454	PEF	5.97 (−6.53 to 18.46)
	FEV_1_	0.01 (−0.07 to 0.09)

O_3_ (40 ppb), *n* = 454	PEF	0.47 (−12.48 to 13.42)
	FEV_1_	0.00 (−0.08 to 0.08)

a From 5th to 95th percentile of pollutant concentration weekdays only, 0900 hours to 2100 hours.

b Same-day afternoon lung function measurements.

c PEF measured in L/min.

d FEV_1_ measured in L.

* *p* < 0.10 by *t*-test.
